# Effects of the TLR2 Agonists MALP-2 and Pam_3_Cys in Isolated Mouse Lungs

**DOI:** 10.1371/journal.pone.0013889

**Published:** 2010-11-16

**Authors:** Martina Barrenschee, Dennis Lex, Stefan Uhlig

**Affiliations:** Institute of Pharmacology and Toxicology, University Hospital Aachen, RWTH Aachen University, Aachen, Germany; Ludwig-Maximilians-Universität München, Germany

## Abstract

**Background:**

Gram-positive and Gram-negative bacteria are main causes of pneumonia or acute lung injury. They are recognized by the innate immune system via toll-like receptor-2 (TLR2) or TLR4, respectively. Among all organs, the lungs have the highest expression of TLR2 receptors, but little is known about the pulmonary consequences of their activation. Here we studied the effects of the TLR2/6 agonist MALP-2, the TLR2/1 agonist Pam_3_Cys and the TLR4 agonist lipopolysaccharide (LPS) on pro-inflammatory responses in isolated lungs.

**Methodology/Principal Findings:**

Isolated perfused mouse lungs were perfused for 60 min or 180 min with MALP-2 (25 ng/mL), Pam_3_Cys (160 ng/mL) or LPS (1 µg/mL). We studied mediator release by enzyme linked immunosorbent assay (ELISA), the activation of mitogen activated protein kinase (MAPK) and AKT/protein kinase B by immunoblotting, and gene induction by quantitative polymerase chain reaction. All agonists activated the MAPK ERK1/2 and p38, but neither JNK or AKT kinase. The TLR ligands upregulated the inflammation related genes Tnf, Il1β, Il6, Il10, Il12, Ifng, Cxcl2 (MIP-2α) and Ptgs2. MALP-2 was more potent than Pam_3_Cys in inducing Slpi, Cxcl10 (IP10) and Parg. Remarkable was the strong induction of Tnc by MALP2, which was not seen with Pam_3_Cys or LPS. The growth factor related genes Areg and Hbegf were not affected. In addition, all three TLR agonists stimulated the release of IL-6, TNF, CXCL2 and CXCL10 protein from the lungs.

**Conclusions/Significance:**

TLR2 and TLR4 activation leads to similar reactions in the lungs regarding MAPK activation, gene induction and mediator release. Several genes studied here have not yet been appreciated as targets of TLR2-activation in the lungs before, i.e., Slpi, tenascin C, Parg and Traf1. In addition, the MALP-2 dependent induction of Tnc may indicate the existence of TLR2/6-specific pathways.

## Introduction

Toll-like receptors play a critical role in the recognition of pathogens by the innate immune system [1], [2]. Gram-positive bacteria are recognized by TLR2 receptors by their signature lipoproteins/lipopeptides [3]. Among all organs, the lungs have the highest expression of TLR2 receptors [4]. Beyond infection [5]–[7], TLR2-receptors may play a role in several pulmonary diseases including fibrosis [8], asthma [9], lung contusion [10] and acute lung injury [11], as suggested by studies in TLR2-deficient mice. Polymorphisms in the Tlr2 gene have been associated with susceptibility to tuberculosis [12]. TLR2 forms heterodimers with either TLR1 or TLR6 and polymorphisms in these receptors have been associated with atopic asthma [13] and organ dysfunction in sepsis [14]. For some time, lipoteichoic acid (LTA) was considered a relevant TLR2 receptor ligand, and most studies on TLR2 receptors in the lungs have focused on this agent [e.g. in [15]–[18]]. However, because recent evidence indicates that LTA is not a TLR2 receptor ligand [3], now only little is known about the effects of true TLR2 ligands in the lungs. Two well defined ligands that permit studying the functions of TLR2 receptors are the lipopetides Pam_3_CSK_4_ and MALP-2, containing three and two fatty acids, respectively [3].

Macrophage-activating lipopeptide 2 KDa (MALP-2) was originally isolated from *Mycoplasma fermentas* [19] and is now available synthetically. It uses CD36 as a coreceptor and signals by TLR2/TLR6 heterodimers [20]. *In vivo*, MALP-2 causes increased BAL levels of several pro-inflammatory cytokines and chemokines as well as neutrophil and lymphocyte infiltration [21], [22]. In murine precision-cut lung slices (PCLS), it had no effect on cytokine release unless it was applied together with interferon γ, in whose presence it increased TNF and IL-1α release [23], whereas in human PCLS and in human airway epithelial cells MALP-2 or an analogon induced several cytokines and chemokines after 24h [24], [25]. Notably, pulmonary application of MALP-2 improved metastasis [26], vaccination [27] and survival in pneumonia [22].

The synthetic bacterial lipopeptide analogon Pam_3_CysSK_4_ (in the following referred to as Pam_3_Cys) acts on TLR1/TLR2 heterodimers [3] In intact lungs it caused cytokine and chemokine release [28], and in human bronchial epithelial cells (hBE) and human alveolar macrophages it stimulated release of TNF, IL-1, IL-6 and IL-8 [29], [30]. There is evidence that TLR1 and TLR6-receptors are not redundant, since the TLR1_-7202G_ mutation is associated with higher mortality rates in sepsis and hyperresponsiveness towards Pam_3_Cys [14].

Thus, recent evidence is indicating that both MALP-2 [22] and Pam_3_Cys [28] can stimulate cytokine and chemokine release in the lungs. However, as these studies where done *in vivo* the contribution of lung parenchyma versus recruited cells to these responses remains unknown. Furthermore, as these ligands stimulate TLR2-receptors differently, i.e. either TLR2/TLR1 heterodimers (Pam_3_Cys) or TLR2/TRL6 heterodimers, the relative potency and specificity of these agents with respect to pro-inflammatory responses in the lungs is unknown. Therefore, to systematically compare the effects of TLR2/TLR1 vs. TLR2/TLR6 in lung tissue independent of recruited leukocytes, we used isolated blood-free perfused mouse lungs to study the effects of MALP-2 and Pam_3_Cys and compared them to those of the well known TLR4 ligand lipopolysaccharide.

## Results

### Lung physiology

**Figure 1:**
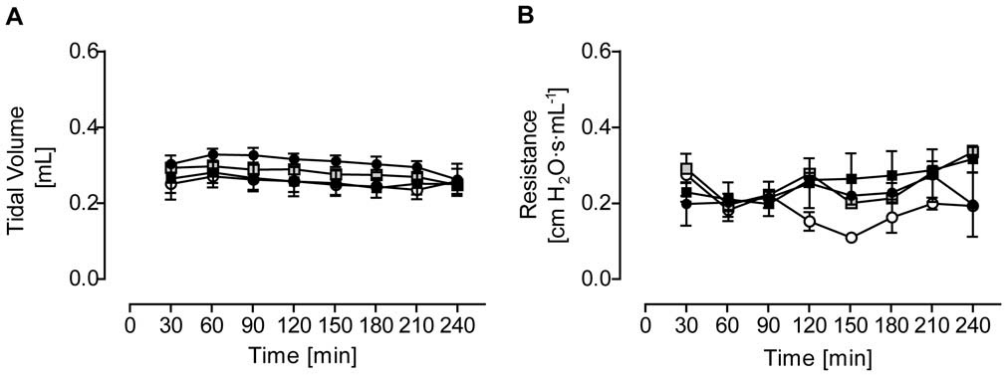
Lung functions. (**A**) Tidal volume and (B) pulmonary resistance in isolated perfused mouse lungs exposed to Pam_3_Cys (160 ng/mL, black squares), MALP-2 (25 ng/mL, grey squares) or LPS (1 µg/mL, black circles) from 60 min to the end of the experiment; control lungs are shown as white circles. Data are expressed as mean ± SEM, n = 3–5.

As reported before [31], LPS administration did not change pulmonary lung functions in isolated perfused mouse lungs (Fig. 1). Likewise, neither MALP-2 nor Pam_3_Cys administration significantly altered tidal volume or pulmonary resistance, their values always being between 0.3 to 0.4 mL and 0.2 to 0.3 cm H_2_O·s·mL^−1^, respectively.

### MAP and Akt kinase

Since TLR2 is well known to activate MAPK pathways [32], we analyzed activation of ERK1/2, JNK and p38. In addition, we also examined phosphorylation of AKT kinase (protein kinase B).

**Figure 2:**
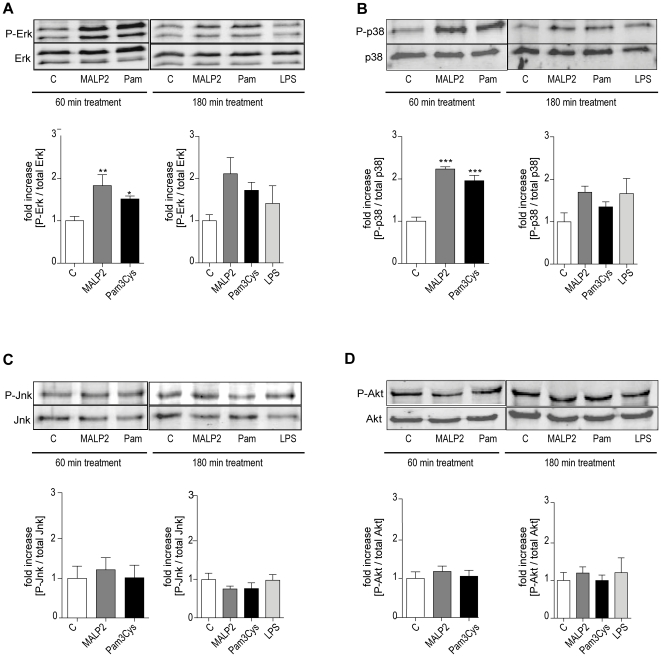
Mitogen activated protein kinase and Akt kinase activation. After 60 min of perfusion under baseline conditions, isolated mouse lungs were perfused for another 60 or 180 min with Pam_3_Cys (160 ng/mL, n = 5), MALP-2 (25 ng/mL, n = 5), LPS (1 µg/mL, n = 3) or under control conditions (n = 5). The basal and phosphorylated forms of several kinases were analyzed by immunoblotting after 60 min or 180 min: (**A**) Erk1/2, (**B**) p38, (**C**) Jnk, and (**D**) Akt kinase. Data were calculated as the ratio of the phosphorylated protein to the total amount of the protein and then referenced to the control on the same gel. Data are shown as mean ± SEM. The micrographs show one representative immunoblot from 3 (LPS) or 5 (C; Pam_3_Cys, MALP-2) independent experiments.

After 60 min, both Pam_3_Cys and MALP-2 increased the phosphorylation of ERK1/2 and p38 compared to controls, whereas JNK and AKT kinase were not affected (Fig. 2). After, 180 min treatment, both TLR2 ligands and also the TLR4 ligand LPS appeared to increase the phosphorylation of p38 and ERK1/2, although these effects were no longer significant. Again, increased phosphorylation of JNK or AKT kinase was not observed.

### Gene expression

We investigated the regulation of 3 genes related to cell growth or differentiation, and an additional 15 genes involved in inflammatory processes. These growth factors and tenascin C (Tnc) were studied because they were found to be upregulated by another potent cause of pulmonary inflammation, i.e. high tidal volume ventilation [33]. The genes were selected for their well known role in a wide variety of inflammatory processes in the lung, i.e. cyclooxygenase 2 (Ptgs2, [34]), poly(ADP-ribose)glycohydrolase (Parg, [35]), secretory leukoprotease inhibitor (Slpi, [36]), Traf1, [37]), IP10 (Cxcl10, [38]), interleukin 1β (Il1b, [39]), MIP-2α (Cxcl2, [40]), interleukin-4 (Il4, [13]), interleukin-6 (Il6, [40]), interleukin-10 (Il10, [41]) interleukin-12 (Il12a, i.e. IL12p35; Il12b,i.e. IL12p40, [42]), interferon γ (Ifng, [43]) and tumor necrosis factor (Tnf, [40]). Gene selection was also based on the distinction that has been made between Myd88-dependent (Tnf, Il1b, Il6), and TRIF-dependent (Traf1, Cxcl10) gene expression [44].

**Figure 3:**
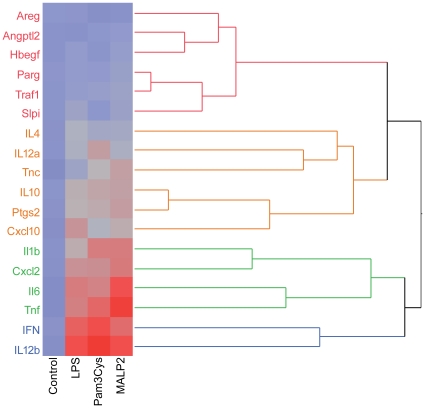
Cluster analysis of the gene expression data. After 60 min of perfusion under baseline conditions, isolated mouse lungs were perfused for another 180 min with Pam_3_Cys (160 ng/mL, n = 5), MALP-2 (25 ng/mL, n = 5), LPS (1 µg/mL, n = 3) or under control conditions (n = 5). The different colors (red, brown, green, blue) identify genes that clustered together.

**Figure 4:**
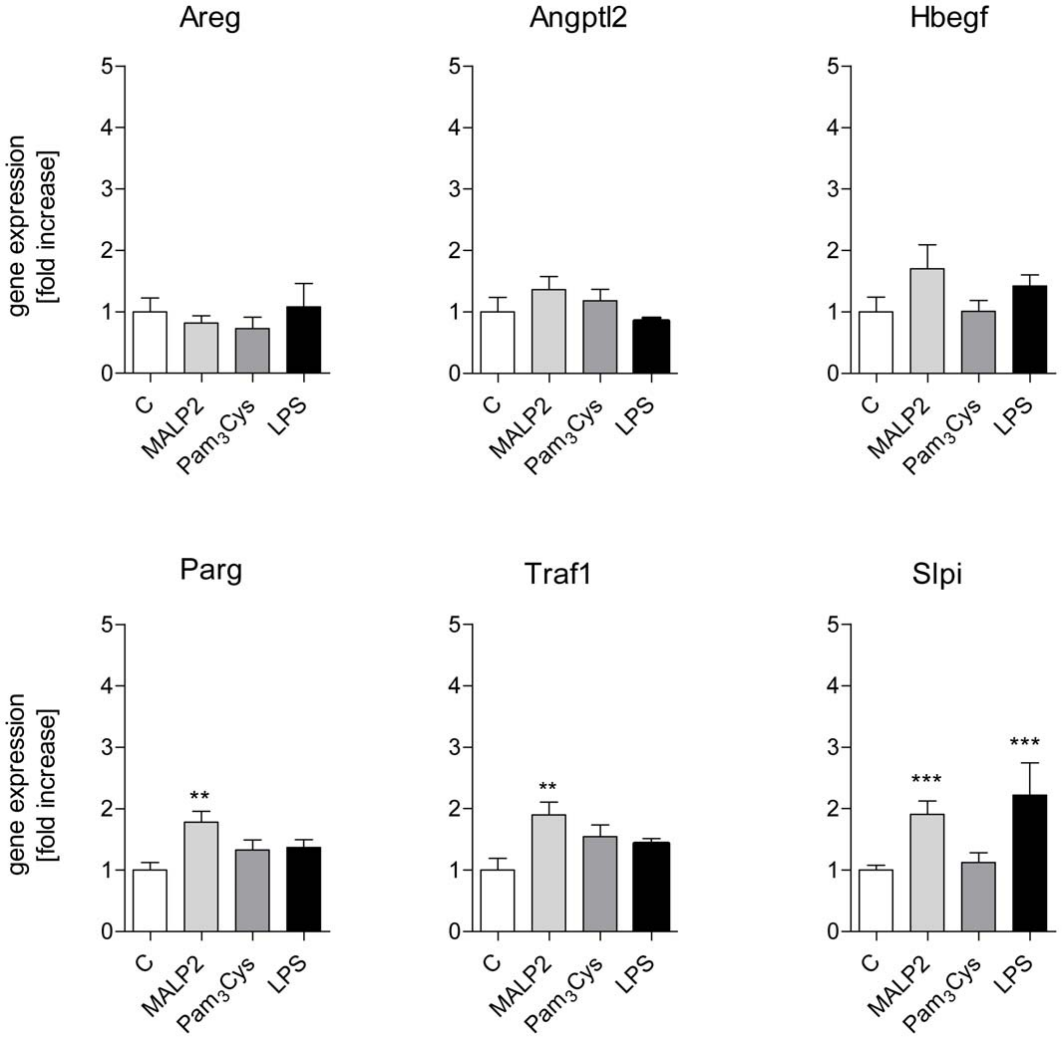
Expression of genes in cluster 1. After 60 min of perfusion under baseline conditions, isolated mouse lungs were perfused for another 180 min with Pam_3_Cys (160 ng/mL, n = 5), MALP-2 (25 ng/mL, n = 5), LPS (1 µg/mL, n = 3) or under control conditions (n = 4). Genes: amphiregulin (Areg), angiopoietin-like 2 (Angptl2), heparin-binding epithelial growth fator (HBegf), poly(ADP-ribose)glycohydrolase (Parg), TNF receptor-associated factor 1 (Traf1), and secretory leukocyte peptidase inhibitor (*Slpi*). Data were normalized to the experimental control and are shown as mean ± SEM. *, p<0.05 vs control; **, p<0.01 vs control; ***, p<0.001 vs control.

The gene expression patterns were analyzed by cluster analysis (Fig. 3) and revealed four major clusters based on the intensity of their expression in response to the different TLR ligands. The data are presented in accordance with these clusters in the figures 4 to [Fig pone-0013889-g005] 6. Please note that the Y-axis is the same in each subpanel of one figure, but differs between the figures.

**Figure 5 pone-0013889-g005:**
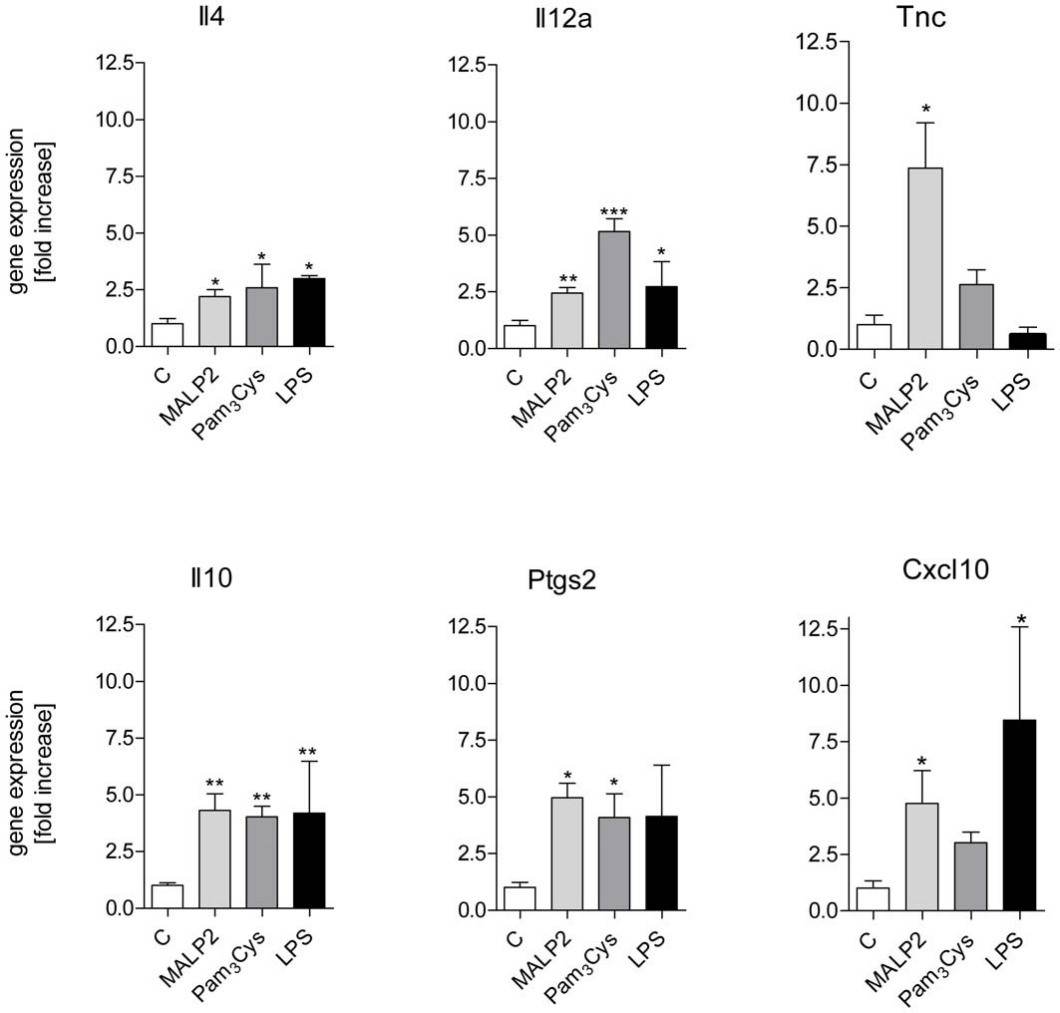
Expression of genes in cluster 2. After 60 min of perfusion under baseline conditions, isolated mouse lungs were perfused for another 180 min with Pam_3_Cys (160 ng/mL, n = 5), MALP-2 (25 ng/mL, n = 5), LPS (1 µg/mL, n = 3) or under control conditions (n = 5). Genes: interleukin 4 (Il4), interleukin 12p35 (Il12a), tenascin C (Tnc), interleukin 10 (Il10), cyclooxygenase 2 (*Ptgs2*), and IP10 (*Cxcl10*). Data were normalized to the experimental control and are shown as mean ± SEM. *, p<0.05 vs control; **, p<0.01 vs control; ***, p<0.001 vs control.

**Figure 6:**
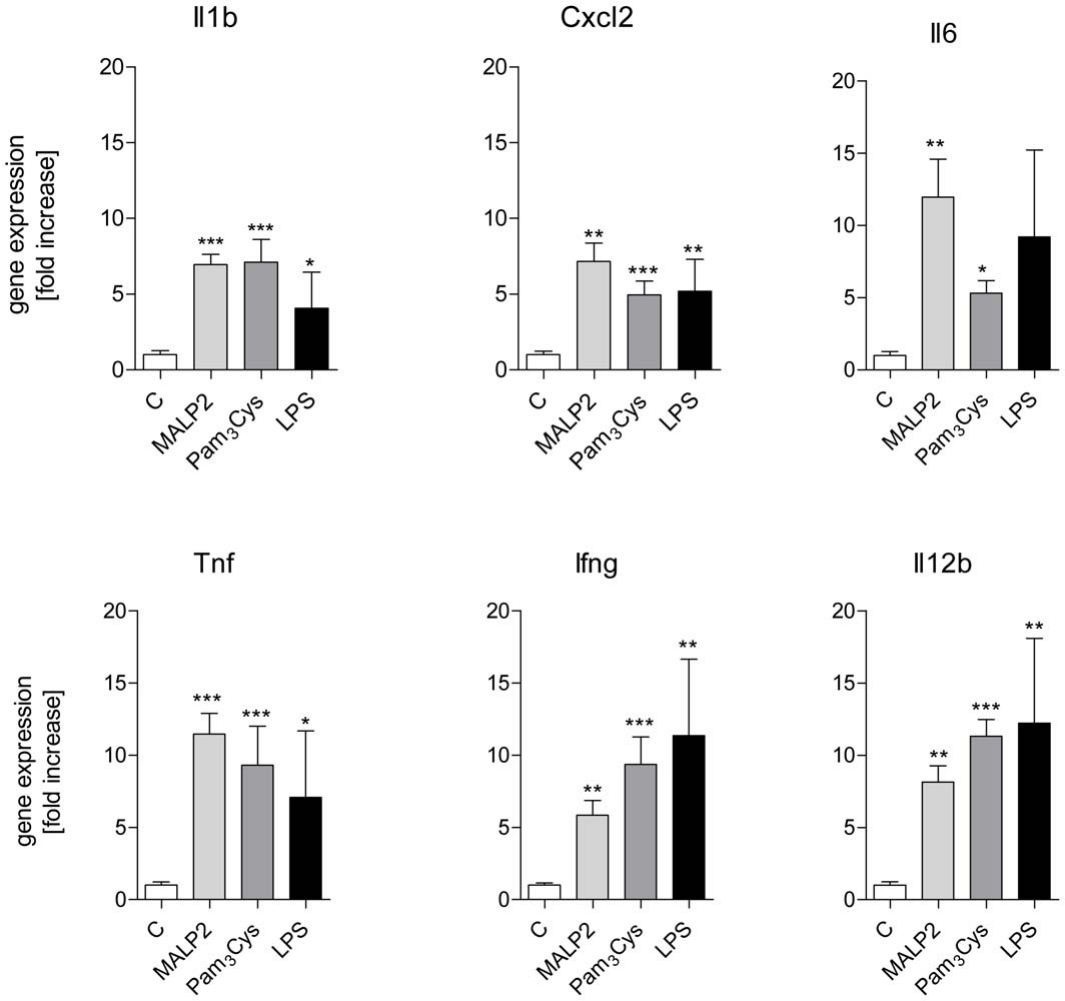
Expression of genes in cluster 3 and 4. After 60 min of perfusion under baseline conditions, isolated mouse lungs were perfused for another 180 min with Pam_3_Cys (160 ng/mL, n = 5), MALP-2 (25 ng/mL, n = 5), LPS (1 µg/mL, n = 3) or under control conditions (n = 5). Genes: interleukin 1β (*Il1b*), macrophage inflammatory protein 2α (*Cxcl2*), interleukin 6 (Il6), tumor necrosis factor (*Tnf*), interferon γ (Ifng), and IL12p40 (IL12b). Data were normalized to the experimental control and are shown as mean ± SEM. *, p<0.05 vs control; **, p<0.01 vs control; ***, p<0.001 vs control.

Cluster 1 (red in Fig. 3, Fig. 4): No or only weakly increased expression. This cluster contained the growth factors angiopoietin-like 2, HB-EGF and amphiregulin that remained unaltered, Parg and Traf1 that were about two-fold upregulated by MALP-2 and Slpi that was about two-fold upregulated by MALP-2 and LPS, but not by Pam3Cys.

Cluster 2 (brown in Fig. 3, Fig. 5). This cluster contained genes that were upregulated between 2.5-fold and about 10-fold. Il4, Il12a, Il10 and Ptgs2 were upregulated by all TLR ligands; Cxcl10 was upregulated by both MALP-2 and LPS, and Tnc was strongly upregulated by MALP-2 alone.

Group 3 and 4: (green and blue in Fig. 3, Fig. 6). This cluster contains genes for inflammatory proteins, that were strongly (>5-fold) upregulated under all conditions. These genes, i.e. *Il1b*, Cxcl2, Il6, Tnf, Ifng and IL12p40 (IL12b) showed comparably strong mRNA expression levels (between 5–15 fold increases) in lung tissue upon TLR2 or TLR4 receptor stimulation, indicating that the concentrations chosen were about equipotent with respect to their pro-inflammatory capacity (Fig. 6).

With respect to differences between MALP-2 and Pam3Cys we noted that Cxcl10 and Slpi were strongly increased by MALP-2 and LPS, but comparatively little by Pam_3_Cys. Three genes were significantly stimulated by MALP-2 only, i.e. Tnc, Parg and Traf1.

### Cytokine release

**Figure 7:**
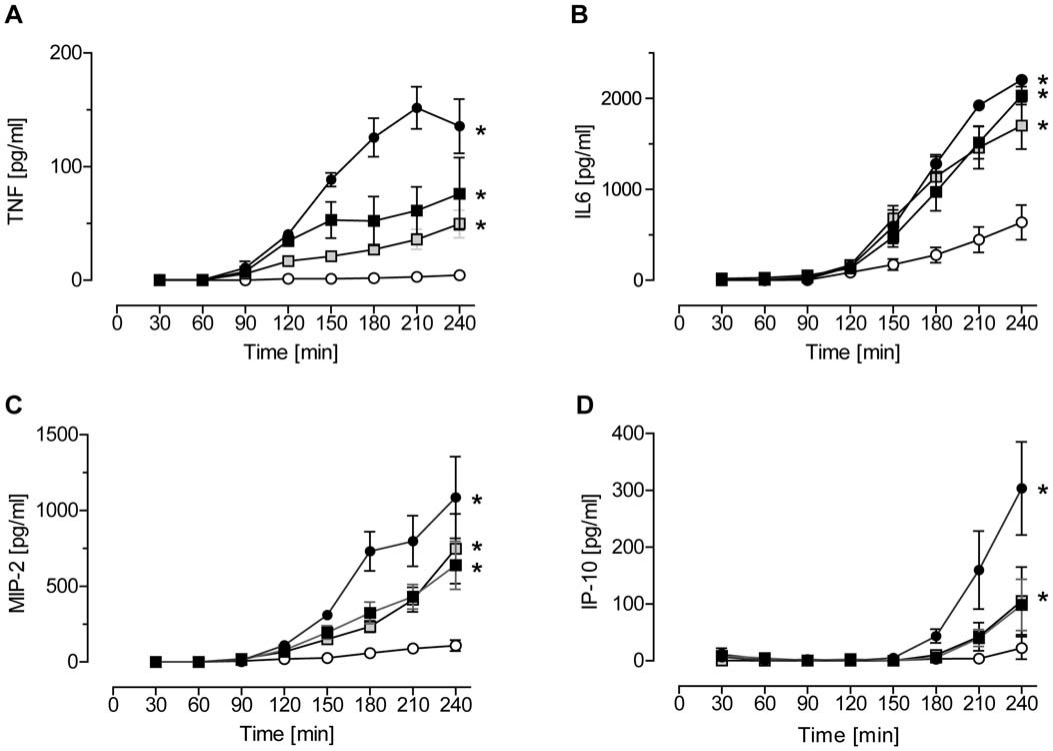
Mediator release into the perfusate. After 60 min of perfusion under baseline conditions, isolated mouse lungs were perfused for another 180 min with Pam_3_Cys (black squares, 160 ng/mL, n = 5), MALP-2 (grey squares, 25 ng/mL, n = 5), LPS (black circles, 1 µg/mL, n = 3) or under control conditions (white circles, n = 4). Perfusate samples were taken every 30 min and analyzed by ELISA for TNF (**A**), IL-6 (**B**), MIP-2α (**C**) and IP-10 (**D**). Data are expressed as mean ± SEM. *, p<0.05 vs. control.

The time course of the release of two cytokines (IL-6, TNF) and two chemokines (MIP-2α, IP10) into the perfusate was measured by ELISA (Fig. 7). As all lungs were perfused with 1 mL/min in a non-recirculating fashion, the concentrations given on the ordinate represent the pulmonary production within 1 minute. The IL-6 production was comparable between all three TLR-ligands. For TNF, MIP-2α and IP10, LPS was always the most potent stimulus, but the TLR2 agonists were effective as well. The time course of release was the same for IL-6, TNF and MIP-2α with production setting in about 60 min after addition of the stimulus. IP-10 production did not start before 120 min after addition of the stimulus.

## Discussion

Gram-positive bacteria present a severe health burden and are a major cause of community acquired pneumonia [45] and acute lung injury [14], [46]. Hence it may not be a coincidence that among all organs the lung is the one with the highest expression of the TLR2, the receptor for recognition of Gram-positive bacteria [4]. Here, we compared the responses of isolated lungs to stimulation of TLR2/TLR1, TLR2/TLR6 and TLR4 receptors by using highly purified ligands in order to exclude unspecific effects. Several genes studied here have not yet been appreciated as targets of TLR2-activation in the lungs before, i.e. Slpi, tenascin C, poly(ADP-ribose)glycohydrolase and Traf1. Our findings suggest that all three receptor systems cause similar responses in the lungs with respect to MAP kinase activation, gene induction and mediator release. Of note, TLR2-ligation induced both Myd88-dependent (TNF, IL-1β, IL-6) and TRIF-dependent (Traf1, IP10) genes. In contrast to the other genes, tenascin C was upregulated by MALP-2, but not by LPS, suggesting the possibility of TLR2-specific signaling pathways in the lungs. Because of perfusion with blood-free buffer, all these responses occurred independent of sequestered leukocytes. On the other hand, the failure of the TLR ligands to cause overt changes in tidal volume or airway resistance supports the notion that effects on physiological lung functions *in vivo* depend on the sequestration of leukocytes.

The present observations on the gene induction and production of IL-6, MIP-2α and TNF confirm previous findings in mouse lungs perfused with LPS [33], [40] and indicate that TLR2 receptor activation leads to similar responses as that of TLR4 receptors. Together with IL-1β these cytokines represent typical genes that are activated by the Myd88-dependent signaling pathway [44], although MIP-2α may also be activated by TRIF [47]. The activation of Myd88-dependent pathways is also supported by the activation of Erk and p38, similar to previous findings in airway epithelial cells, alveolar macrophages [48], [49] and intact lungs [50]. Corroborating results with LPS [51], TLR2 ligands failed to activate Akt in isolated lungs; thus the activation of Akt that is caused by LPS *in vivo* [50] is probably related to the phosphorylation of Akt in sequestered leukocytes [52].

The increase in IP10 gene and protein levels by TLR2 ligation (Fig. 5, Fig. 7,[24]) – although not as strong as with LPS – is somewhat unexpected, since IP-10 is typically considered to depend on TRIF which according to present knowledge does couple to TLR4 but not TLR2 receptors. In addition, MALP-2 was even stronger than LPS in inducing another TRIF-dependent gene, namely Traf1. As an explanation, contamination of the MALP2 with LPS seems unlikely, because both MALP-2 and Pam_3_Cys were synthetically produced and perfused at concentrations several times lower than LPS – concentrations at which LPS shows little effect in our system [31; and unpublished observations]; furthermore, it is well known that in the nanomolar range LPS is inactive in the absence of lipopolysaccharide binding protein [53]. More likely explanations for the activation of IP10 and Traf1 by TLR2 agonists are: (i) The production of IP10 started later than that of the other cytokines; thus it seems possible that IP10 was induced by other cytokines such as IL-1β, TNF or IFNγ [38]. (ii) There might exist some small Myd88-dependent part in MALP-2-induced activation of IP10 [54] or even another TRIF-independent pathway that is activated by MALP-2; (iii) Many studies on the role of TRIF in the expression of IP10 or Traf1 have been performed in myeloid or fibroblast cells in culture. However, TRIF expression is particularly abundant in the lungs [4], and there are cell-type and organ-specific differences in the regulation of TLR-receptor signaling [55], [56]. In fact, MALP-2-dependent release of IP10 had been observed before in human airway epithelial cells in culture [24]. Of interest, in another recent study Pam_3_Cys was able to strongly induce RANTES – another TRIF-dependent cytokine – in human airway epithelial cells [48]. Collectively, these studies suggest that the distinction between Myd88-dependent and TRIF-dependent genes is not helpful to predict mediator production from intact organs that consist of many different cell types. Nonetheless, in future studies it would be of great interest to learn which pulmonary cell type is responsible for the production of specific cytokines *in situ*.

Because TLR1 and TLR6-receptors do not appear to be redundant [14], one aim of the present study was to compare the consequences of TLR2/TLR1 *vs*. TLR2/TRL6 receptor activation in the lungs. Therefore, we have employed Pam_3_Cys that signals through TLR2/TLR1 heterodimers and MALP-2 that signals through TLR2/TRL6 receptors. The concentrations required for pro-inflammatory signaling were relatively low for perfusion experiments in intact lungs, i.e. 25 ng/mL for MALP-2 and 160 ng/mL for Pam_3_Cys. At these concentrations, both TLR2 ligands showed a strong pro-inflammatory response. Overall the responses to both TLR2 ligands on MAP kinases, gene expression and mediator production were similar. However, MALP-2 was able to induce the expression of three genes that were not affected by Pam_3_Cys, i.e. Slpi, Tnc and Parg. As these genes had not before been studied following TLR2 receptor activation, the significance of these findings remains to be shown. Others have reported in candidiasis that TLR1 deficient mice had no altered phenotype, whereas TLR6-deficient mice produced similar levels of TNF, IL-1 and IL-6, but less IFNγ and IL-10, providing further evidence that some genes may be regulated specifically by TLR2/TLR6-receptor heterodimers [57], although from thorough studies in cell culture it was concluded that TLR1 and TLR6 lead to identical signaling events [58].

The pattern of gene transcription was very similar between the three TLR-receptor agonists studied and all of them induced several genes that are known to be involved in inflammation (see Fig. 5,6). It should also be noted that agonists of TLR-ligands such as the agents that were studied here, may amplify their own responses by upregulation of TLR-receptors [59]–[61], although the pathophysiological significance of this response in the lungs remains to be further established [62], [63]. Two observations may require further comments. First, the growth factor ligands amphiregulin (*Areg*) and heparin-binding EGF-like growth factor (*Hbegf*) that are well known to be activated by mechanical stimuli [33], [64], were not affected by TLR2 or TLR4-receptor activation. This is noteworthy, because otherwise most of the genes that are activated by LPS in the lungs are also activated by mechanical ventilation [33], [65]–[67], suggesting that amphiregulin and HB-EGF may be used as molecular markers to distinguish infectious from mechanical stimuli. Second, tensascin C, an extracellular matrix (ECM) glycoprotein within the tenascin familiy [68] was highly upregulated by MALP-2 (Fig. 4). Tenascin C harbors an EGF-like domain [68], and shows low but detectable action on the EGF-receptor [69]. It is highly expressed in lungs from patients with respiratory distress syndrome and bronchopulmonary dysplasia, although its role in the pathogenesis of these ailments is unknown [70]. In general, tenascins are thought to be involved in tissue remodeling after injury [71], providing a possible explanation for the beneficial effects of MALP-2 on wound healing in mice [72] and humans [73].

In conclusion, TLR1/2-, TLR 2/6- and TLR4-specific ligands induced Th1-cytokines (Tnf, Ifng, Il12), Th2 cytokines (Il4) and anti-inflammatory genes (Slpi, Il10). Despite the fact that TLR2-receptors do not couple to TRIF, the effects of TLR2/1, TLR2/6 and TLR4-receptor activation in the isolated lungs on MAPK activation, gene induction and cytokine release were largely similar. In addition to several well known pro-inflammatory cytokines we also demonstrate TLR2-dependent production of IP10, a chemokine which is upregulated in acute lung injury [74], although its functions in this setting remain poorly defined. Other genes shown to be upregulated by TLR2-receptor activation were Slpi, tenascin C, poly(ADP-ribose)glycohydrolase and Traf1. Particularly impressive and restricted to MALP-2 (TLR2/6) was the induction of tenascin C suggesting the existence of TLR2/6-specific pathways.

## Materials and Methods

### Animals and reagents

Female BALB/C mice were obtained from Harlan Laboratories (The Netherlands). All animals were used at a weight of 20 to 25 g. Care and handling of the animals was performed in accordance with the regional committee of animal experimentation ethics.

HES-buffer for isolated perfused mouse lungs was custom-made by SERAG-Wiessner (Naila, Germany). Primary antibodies were obtained from Cell Signaling Technology CST (Frankfurt, Germany), the secondary ones (goat-anti mouse IRDye800CW, goat-anti rabbit IRDye 680) for fluorescence detection with the ODYSSEY-System (LI-COR, Bad Homburg) were purchased from LI-COR. Primers for RT-qPCR were synthesized and purified by MWG (Ebersberg, Germany). All other chemicals and substances used were of analytical grade and commercially available. Pam_3_CysSK4 was from EMC microcollections (Tübingen, Germany), MALP-2 from Alexis Biochemicals (Grünberg, Germany). Highly purified lipopolysaccharide from S. *abortus equii* [75] was kindly provided by Helmut Brade (Research Center Borstel, Borstel, Germany).

### Isolated perfused mouse lung preparation (IPL)

The mouse lungs were prepared and perfused as described [76], [77], with small modifications in the buffer (4% hydroxyl ethyl starch instead of 4% BSA).

All lungs were perfused at 1 mL/min and ventilated for 60 min under control conditions with an end-inspiratory pressure (EIP) of -10 cm H_2_O and an end-expiratory pressure (EEP) of -3 cm H_2_O, resulting in tidal volumes of about 300 µL and perfused and stimulated either for another 60 min (experimental set 1) or 180 min (experimental set 2).

Set 1: After 60 min, the lungs were randomly allocated to one of the following four groups and perfused and ventilated for another ***60 min***: group 1 under control conditions, group 2 with addition of 160 ng/mL (100 nM) Pam_3_CysSK_4_ and group 3 with 25 ng·mL^−1^ MALP-2.

Set 2: After 60 min, the lungs were randomly allocated to one of the following four groups and perfused and ventilated for another ***180 min***: group 1 under control conditions, group 2 with 160 ng/mL Pam_3_CysSK_4_, group 3 with 25 ng/mL MALP-2, group 4 with 1 µg/mL lipopolysaccharide (LPS) from *S. abortus equii*. After ventilation, the surrounding tissue, the heart, and the trachea were trimmed away, subsequently lungs were flash frozen under liquid nitrogen and stored at -80°C.

### Immunoblotting

Aliquots of 30 mg lung powder were lysed and homogenized in cell extraction buffer (Biosource), containing 1 mM PefaBlock (Roche-Diagnostics) and complete mini (Roche-Diagnostics) according to manufacturer's instructions.

After 30 min on ice, lysates were collected by pelleting the cellular debris for 10 min at 16.000× *g*. Total protein content was determined by BCA Protein Assay Kit (Pierce, Rockford, USA). Equal amounts of protein (30 µL/slot) were size fractionated by sodium dodecyl sulphate (SDS)-polyacrylamide gel electrophoresis, transferred to nitrocellulose transfer membranes (Protran, Schleicher & Schuell, Dasel,Germany) and immunoblotted with primary antibodies that are specific for the phosphorylated and non-phosphorylated form of the investigated protein (Cell Signaling Technology CST (Frankfurt, Germany).

 After incubating and washing, nitrocellulose membranes were incubated with both secondary fluorescence labelled antibodies (LI-COR, Bad Homburg). Detection and quantification was realized using the Odyssey® infrared imaging system by LI-COR (Lincoln, Nebraska USA). Protein bands were pictured at 700 nm and 800 nm simultaneously in a single scan, using the Odyssey Infrared Imaging system (LICOR, Bad Homburg, Germany). Specific protein bands were quantified with Odyssey imaging software. Integrated intensities of specified bands were expressed as ratio of the phosphorylated to the non-phosphorylated form of the detected protein; protein expression is expressed as fold increase relative to controls.

### Reverse transcriptase quantitative real time PCR (RT-qPCR)

Total RNA from 30 mg lung powder were isolated with NucleoSpin® RNA II Kit according to manufacturer's instructions (Machery Nagel, Düren, Germany). 8 µL total RNA was completed with 2 µL Oligo-(dt)-primer (Invitrogen, Karlsruhe,Germany), mixed, centrifuged and subsequently incubated at 65°C for 10 min to linearize RNA. 2 µL of this RNA-primer-mix were added to 1 µL Superscript II™ Reverse Transcriptase (200 U/µL) (Invitrogen), 4 µl 5x Strand Puffer (250 mM Tris/HCl, ph 8.3; 375 mM KCl,15 mM MgCl_2_) (Invitrogen), 2 µL DTT (0.1M) (Invitrogen), 2 µl dNTP-Mix (10 mM) (peqLAB, Erlangen), 1 µL RNaseOut (40 U/µL) (Invitrogen) and incubated for 90 min at 37°C. Afterwards, the mixture was diluted with 30 µL H_2_O and real-time qPCR product accumulation was monitored in a Light-Cycler480 (Roche-Diagnostics) using 1 µL of total cDNA, 312.5 nM forward, 312.5 nM reverse primer and SYBR-Green I Mastermix (Roche-Diagnostics, Mannheim, Germany) according to manufacturer's instructions. The primers are shown in Table 1. The data were normalized first to the Tyk2 gene (see next paragraph); gene expression is expressed as fold induction relative to controls.

**Table 1:** Primer for mouse-target genes used in quantitative-real-time PCR (qPCR).

target gen	primer (sense)	primer (antisense)
*Angptl2*	*5′-GAGAATACCAACCGCCT-3′*	*5′- ATAGGTCTCCCAGTTCC-3′*
*Hbegf*	*5′-GTGTTGTCCGCGTTGGT-3′*	*5′- TGTCCCTTCCAAGTCCT-3*
*Areg*	*5′-CTATCTTTGTCTCTGCCATCA-3′*	*5′-AGCCTCCTTCTTTCTTCTGTT-3′*
*Parg*	*5′-GTGACTGTTCGGGTAGAC-3′*	*5′- GTTCGCTCACCATTCTCATC-3′*
*Traf1*	*5′-TGAGAACCTGAGAGATGATG-3′*	*5′- TGAAGGAACAGCCAACACC-3′*
*Slpi*	*5′-TGAGAAGCCACAATGCCG-3′*	*5′- CACTGGTTTGCGAATGGG-3′*
*Cxcl10*	*5′-GCCGTCATTTTCTGCCTCAT-3′*	*5′-GCTTCCCTATGGCCCTCATT-3′*
*Ptgs2*	*5′-AGATGACTGCCCAACTCCCAT-3′*	*5′-CAGGGATGAACTCTCTCCGTA-3′*
*Tnc*	*5′-CTTCATTCGTGTGTTCGCCA-3′*	*5′-ATCCCACTCTACTTCCACAG-3′*
*Il1b*	*5′-GAAAGCTCTCCACCTCAATG-3′*	*5′-GCCGTCTTTCATTACACAGG-3′*
*Cxcl2*	*5′-AGTGAACTGCGCTGTCAATGC-3′*	*5′-AGGCAAACTTTTTGACCGCC-3′*
*Il6*	*5′-CCAGAGATACAAAGAAATGATGG-3′*	*5′-ACTCCAGAAGACCAGAGGAAA-3′*
*Tnf*	*5′-TCTCATCAGTTCTATGGCCC-3′*	*5′-GGGATGAGACAAGGTACAAC-3′*
*Tyk2*	*5′-AGTGTTCTGGTATGCCC-3′*	*5′- TGGTTAGAGTCACAGTATG-3′*
*B2m*	*5′-TGACCGGCTTGTATGCTATC-3′*	*5′-CAGTGTGAGCCAGGATATAG-3′*
*Rp32*	*5′-AGCGAAACTGGCGGAAAC-3′*	*5′- GACCAGGAACTTGCGGAA-3′*
*Hprt1*	*5′-TTATGGACAGGACTGAAAGA-3′*	*5′-TGTAATCCAGCAGGTCAGCA-3′*
*Ifng*	*5′-GAGGTCAACAACCCACAGGTC-3′*	*5′- CGAATCAGCAGCGACTCCT-3′*
*Il10*	*5′-GAAGACCCTCAGGATGCG-3′*	*5′-GCCTTGTAGACACCTTGGTC-3′*
*Il12a*	*5′-TGTCAATCACGCTACCTCCTC -3′*	*5′-TCGGGACTGGCTAAGACAC-3′*
*Il12b*	*5′-CAAGAGCAGTAGCAGTTCCC-3′*	*5′-GGTCCAGTGTGACCTTCTCT-3′*
*Il4*	*5′-GTCATCCTGCTCTTCTTTCTC-3′*	*5′-TCTCTGTGGTGTTCTTCGT-3′*

**Figure 8:**
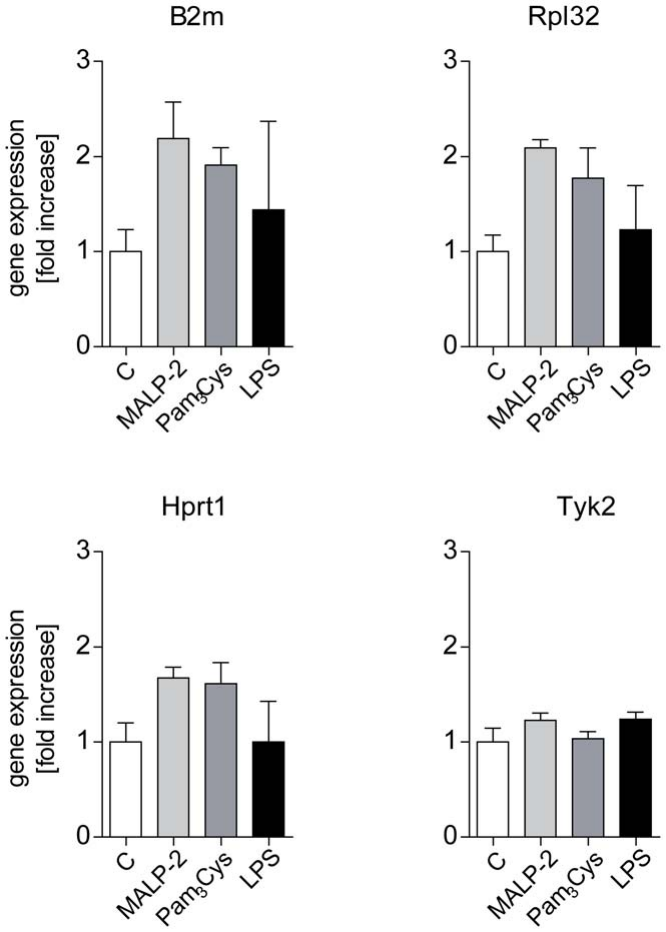
Reference genes for RT-qPCR experiments. After 60 min of perfusion under baseline conditions, isolated mouse lungs were perfused for another 180 min with Pam_3_Cys (160 ng/mL, n = 5), MALP-2 (25 ng/mL, n = 5), LPS (1 µg/mL, n = 3) or under control conditions (n = 4). Shown are four potential callibrator genes: β2-microglobulin (b2m), ribosomal protein L 32 (rpl32), hypoxhantine phosphoribosyl transferase I (hprt1) and tyrosine kinase 2 (tyk2). Data were normalized to the experimental control and presented as mean ± SEM.

Reference genes, often also referred to as calibrator genes are used to normalize RNA levels between different samples. However, since their expression level may vary among tissues, cells and treatment conditions, the selection of a useful reference gene is critical [78]. In our studies in isolated and ventilated perfused mouse lungs, we investigated the expression of three commonly used housekeeping genes β2 microglobulin (B2m*),* ribosomal protein L 32 (Rpl32) and hypoxhantine phosphoribosyl transferase I (Hprt1), and in addition also of tyrosine kinase 2 (Tyk2). Among these, only tyrosine kinase 2 (Tyk2) did not differ between the treatment groups and was therefore chosen as the reference gene for RT-qPCR (Fig. 8).

### Cytokine concentration in the perfusate

For detection of TNF, IL-6, MIP-2α and IP-10 in perfusate samples, commercially available tests (OptEIATM Set Mouse TNF (mono/poly), OptEIATM Mouse IL-6 Set, BD Biosciences, San Diego, CA, USA, Quantikine Mouse MIP-2 Immunoassay, R&D Systems GmbH, Wiesbaden, Germany and the CXCL10 DuoSet ELISA Kit, R&D Systems) were purchased and performed exactly according to the manufacturer's instructions.

### Statistics

Data were analyzed with JMP 7.0.1 for windows, and expressed as mean ± standard error (SEM). Data were always transformed by the Box-Cox transformation and examined by two-sided student's t-test. Homoscedasticity was confirmed by the Levene-test. P-values were corrected for multiple comparisons according to the false-discovery rate procedure, using R 2.8.0 [33]. p<0.05 vs control was considered significant. Cluster analysis were performed in JMP 7.0.1, using the Complete clustering method with normalized RT-qPCR data.
